# Smoking-mediated nicotinic acetylcholine receptors (nAChRs) for predicting outcomes for head and neck squamous cell carcinomas

**DOI:** 10.1186/s12885-022-10161-x

**Published:** 2022-10-25

**Authors:** Yujie Shen, Qiang Huang, Mengyou Ji, Chi-Yao Hsueh, Liang Zhou

**Affiliations:** Department of Otorhinolaryngology Head and Neck Surgery, Eye, Ear, Nose, and Throat Hospital, Fudan University, Shanghai, China

**Keywords:** Smoking, nAChRs, HNSCC, Prognosis, Outcome

## Abstract

**Background:**

As a human tumor disease, head and neck squamous cell carcinoma (HNSCC) is associated with a high mortality rate worldwide. Nicotinic acetylcholine receptors (nAChRs) are transmembrane receptor proteins and exert their biological effects following activation by nicotine. We aimed to construct a prognostic signature based on the expression of nAChRs among smokers with HNSCC.

**Methods:**

The transcriptome profile of nAChRs was obtained from The Cancer Genome Atlas (TCGA). Following the integration of survival information, univariate Cox regression and least absolute shrinkage and selection operator (LASSO) analyses were performed to screen the prognosis-related nAChRs and construct a prognostic signature. Kaplan–Meier (KM), receiver operating characteristic (ROC), principal component analysis (PCA), and independent prognostic analysis were utilized to verify the predictive power of the nAChR-associated prognostic signature. The expression of α5 nAChR in clinical samples was verified by quantitative reverse transcriptase PCR.

**Results:**

Subunits α2, α5, α9, and β4 were related to the prognosis. The prognostic signature comprised the expression of subunits α5, α9, and β4. The nAChR-associated signature showed high sensitivity and specificity for prognostic prediction and was an independent factor for overall survival. Based on the clinical variables and expression of nAChRs, a nomogram was constructed for predicting the outcomes of HNSCC patients who were smokers in the clinical settings. In clinical specimens, α5 nAChR showed high expression in HNSCC tissues, especially among smokers.

**Conclusions:**

The nAChR-associated signature constructed in this study may provide a better system for the classification of HNSCC patients and facilitate personalized treatment according to their smoking habits.

**Supplementary Information:**

The online version contains supplementary material available at 10.1186/s12885-022-10161-x.

## Background

Head and neck squamous cell carcinoma (HNSCC) frequently originates in the oral cavity, larynx, or pharynx, and is the most common histopathological type of head and neck cancer [1]. Unhealthy lifestyles, including smoking and alcohol consumption, are major risk factors for HSNCC, especially cancers of the oral cavity and larynx [1]. Oropharyngeal cancer is closely associated with human papillomavirus (HPV) infection [2]. Accumulating evidence links HNSCC to alcohol consumption and HPV infection; however, smoking remains the most prominent risk factor for head and neck cancer, accounting for 70% of the cases of HNSCC [3–5]. Surgery, radiotherapy, and cisplatin-based chemotherapy are common treatment strategies for HNSCC [6]. Despite the continuous improvement in the treatment strategies for HNSCC, the incidence and mortality rate increase annually [7]. Thus, there is a need to construct an effective signature to assist in early diagnosis and identify high-risk HNSCC patients with poor outcomes, especially among smokers.

Nicotinic acetylcholine receptors (nAChRs) are transmembrane receptor proteins, important for neuronal signaling relying on the neurotransmitter, acetylcholine [8]. Tobacco burning releases nicotine which activates nAChRs and increases their permeability to calcium and sodium ions [9]. To date, 16 human nAChRs including subunits α1-α7, α9, α10, β1-β4, γ, δ, and ε have been discovered in muscles and neural tissues [10]. Subunits α1, β1, γ, δ, and ε show prominent expression in muscle while the expression of α2-α7, α9, α10, and β2-β4 is high in other non-neuronal tissues [11]. The impact of smoking on human body has been elucidated given the role of nAChRs.

Despite the excellent progress in our understanding of smoking-associated diseases over the years, the relationship between smoking-mediated nAChRs and HNSCC remains largely unclear. In the present study, we integrated the transcript profiles of nAChRs and constructed a novel nAChR-associated signature which was found to have high predictive power amongst HNSCC patients who were smokers.

## Materials and methods

### Acquisition and differential analysis of nAChR expression profile

To investigate the prognosis of the smoking population among HNSCC patients, the mRNA expression profiles of nAChRs for 410 HNSCC samples who were smokers, including 378 tumor and 32 adjacent normal tissues, were extracted from The Cancer Genome Atlas (TCGA, https://portal.gdc.cancer.gov) [12] for differential expression analysis. The clinical information on the smoking population of HNSCC patients is provided in Supplementary Table 1. The clinical information of the 32 adjacent normal tissues is presented in Supplementary Table 2. The Wilcoxon Rank-Sum Test was used to assess differential nAChR expression between tumor and normal tissues, wherein significance was defined as *p < 0.05, **p < 0.01, and ***p < 0.001.

### Protein–protein interactions and pathway enrichment analysis

To examine the criticality of nAChRs in protein interactions, we imported the nAChRs into the STRING database (https://string-db.org/) [13] and visualized the network on Cytoscape (Version 3.7.1). In the network, the size of the node represented the degree value while the thickness of the edge represented the confidence of the experimental evidence. Functional analysis was performed to review the biological processes of nAChRs using the Kyoto Encyclopedia of Genes and Genomes (KEGG) [14] algorithm.

### Construction of a prognostic signature based on nAChR expression

Univariate Cox regression analysis was performed to screen the prognosis-related nAChRs among the smoking population in the HNSCC cohort. To avoid overfitting, least absolute shrinkage and selection operator (LASSO) analysis was performed, and a novel prognostic signature was constructed using the following formula: risk score = nAChR (A) expression * coef (A) + nAChR (B) expression * coef (B) + nAChR (i) expression * coef (i) [15]. Based on the expression of nAChRs, the risk of each of the patients was scored and they were divided into low- and high-risk groups according to the median value. Kaplan–Meier (KM) method, receiver operating characteristic (ROC), and principal component analysis (PCA) were performed to verify the predictive power of the above-described prognostic signature.

### Immune infiltration and drug sensitivity analysis

To investigate the relationship between nAChR-associated signature and immunity, the CIBERSORT algorithm [16] was used to calculate the abundance of 22 immune cell types between the risk groups. We also performed a single-sample gene set enrichment analysis (ssGSEA) to assess the immune activities between the risk groups [17]. The transcript expression profile of each patient was uploaded to the Tumor Immune Dysfunction and Exclusion (TIDE) website (http://tide.dfci.harvard.edu/) and TIDE scores were obtained to predict the immunotherapeutic responses of these patients. This set of scores was compared between low- and high-risk groups. Further, we screened the effective chemotherapeutic drugs targeting the smoking population in the HNSCC cohort using the Genomics of Drug Sensitivity in Cancer (GDSC) database (https://www.cancerrxgene.org/) [18].

### Prognostic analysis for independence and construction of a nomogram

To determine the independent prognostic factors for the smoking population in the HNSCC cohort, a multivariate analysis was performed, considering all the significant prognostic factors obtained from the univariate analysis. Based on the nAChR expression and other clinical variables, a nomogram was constructed using the “rms” package in R software [19], to guide clinical decision-making for the smoking population in the HNSCC cohort. To assess the efficacy of the constructed nomogram, time-dependent calibration curves and ROC curves were plotted and analyzed.

### Tissue specimen collection for quantitative reverse transcriptase PCR (qRT-PCR) analysis

In this study, 61 paired samples of HNSCC were collected from Eye and ENT Hospital, Fudan University between October 2020 and July 2021. These were stored at -80 °C until subsequent qRT-PCR analysis. The Ethical Committees of Eye and ENT Hospital, Fudan University approved the study design (2018036) and informed consent was signed by HNSCC patients. The clinical information of 61 paired samples is provided in Supplementary Table 3. We performed qRT-PCR according to the protocol described in our previous study [20]. We aimed to verify the expression of nAChRs in a different independent cohort and the sequences of all primers used in this study are listed in Supplementary Table 4.

### Public databases

To validate the results of TCGA, we performed a survival analysis based on the expression of α5, α9, and β4 nAChRs in all HNSCC patients using the Kmplot database [21]. In addition, methylated sites of α5, α9, and β4 nAChRs in all HNSCC patients were investigated using the MethSurv database [22]. Also, we performed immune infiltration analysis based on the expression of α5, α9, and β4 nAChRs in HNSCC patients using the Tumor IMmune Estimation Resource (TIMER) database [23].

### Statistical analysis

All statistical analyses were conducted using the R software (version: × 64 3.6.1) and GraphPad Prism 7 and a *p*-value < 0.05 was considered statistically significant.

## Results

### Expression of nAChRs and enriched pathways in the smoking population of the HNSCC cohort

**Fig. 1:**
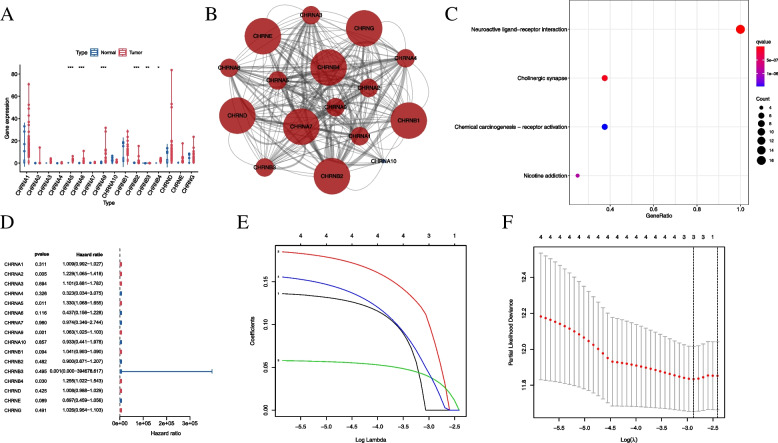
Expression of nAChRs and classification based on the prognosis in the smoking population in the HNSCC cohort. **A** Expression profile of nAChRs; blue and red dots represent normal adjacent and tumor tissues, respectively. *p < 0.05, **p < 0.01, ***p < 0.001. **B** Protein–protein interaction network for nAChRs; circles represent nAChRs; the thickness of the line represents the strength of the experimental evidence while the size of the circle represents the degree value. **C** Significantly enriched signaling pathways based on nAChR expression. **D** Univariate Cox regression analysis for nAChRs; blue and red dots represent protective and risk factors, respectively. **E** LASSO coefficient profiles; nAChRs with non-zero coefficients corresponding to the same log lambda value were selected for the construction of the model, red curve represents CHRNA5, blue curve represents CHRNA9, green curve represents CHRNB4, and black curve represents CHRNA2. (F) LASSO deviance profiles; the log lambda value corresponds to the minimum cross-validation error point

Of all the nAChRs, α5, α6, α9, β2, β3, and β4 were significantly differentially expressed in the smoking population of the HNSCC cohort (Fig. 1A). In addition, the expression profile of β3 nAChR is shown in Supplementary Fig. 1A. As shown in Fig. 1B, nAChRs interact closely with each other evidenced by experimental data, and were significantly overrepresented in neuroactive ligand-receptor interaction, cholinergic synapse, chemical carcinogenesis-receptor activation, and nicotine addiction (Fig. 1C). These results indicated that nAChRs were strongly related to the carcinogenic process and may be potential novel biomarkers for the smoking population in the HNSCC cohort.

### nAChR-based prognostic signature for predicting outcomes in the smoking population of the HNSCC cohort

Four nAChRs, namely α2, α5, α9, and β4, were screened from a univariate Cox regression analysis based on the significance criterion of *p* < 0.05 (Fig. 1D). To avoid overfitting, LASSO regression was performed, and three nAChRs were selected for constructing a prognostic signature (Fig. 1E-F) using the following formula: risk score = α5 nAChR expression * 0.077 + α9 nAChR expression * 0.035 + β4 nAChR expression * 0.029.

**Fig. 2:**
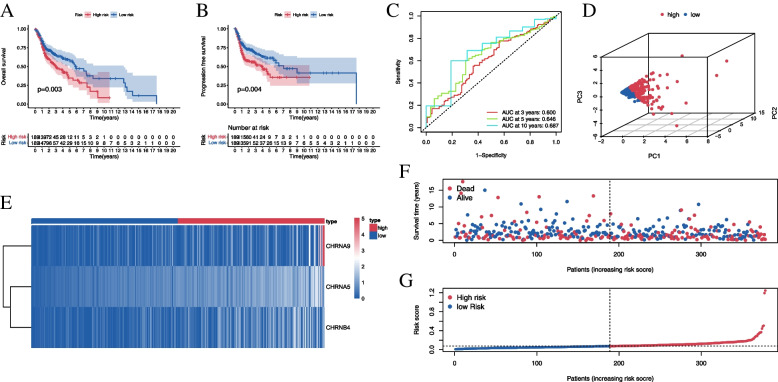
Performance of nAChR-associated signature for prognostic classification. **A** Kaplan–Meier curve for overall survival (OS) suggests that patients in high-risk groups (red curve) show a poorer OS than those in the low-risk group (blue curve); **B** Kaplan–Meier curve for progression-free survival shows that patients in the high-risk groups (red curve) have poorer progression-free survival than those in the low-risk group (blue curve); **C** Time-dependent ROC curve; the AUC values of the nAChR-based signature for three- (red curve), five- (green curve), and ten-year (blue curve) OS are annotated at the bottom right; **D** Principal component analysis shows that the high- (red dots) and low-risk (blue dots) groups were distinguishable based on the formula for the prognostic model; **E** Heatmap for the expression of components of the nAChR-based signature; the abscissa represents HNSCC samples which were divided into high- (red dots) and low-risk (blue dots) groups, and the ordinate represents the expression of nAChRs. The higher the level of expression, the darker the color (red and blue indicate upregulated and downregulated expressions, respectively); **F** Plot for the survival status based on the signature; **G** Risk score plot for the nAChR-based signature

The patients were classified according to their risk scores calculated based on nAChR expression into low- and high-risk groups. Patients in the high-risk group showed poorer overall survival (OS) and progression-free survival (PFS) as compared to those in the low-risk group (Fig. 2A-B). The values of area under the curve (AUC) for nAChR-based signature for three-, five-, and ten-year OS were 0.600, 0.646, and 0.687, respectively (Fig. 2C). The signature showed a great clustering ability according to PCA (Fig. 2D). High levels of expression of the three nAChRs and high mortality were observed in the high-risk group as compared to the low-risk group (Fig. 2E-G).

### Relationship between expression of the prognostic signature and clinicopathological factors

**Fig. 3:**
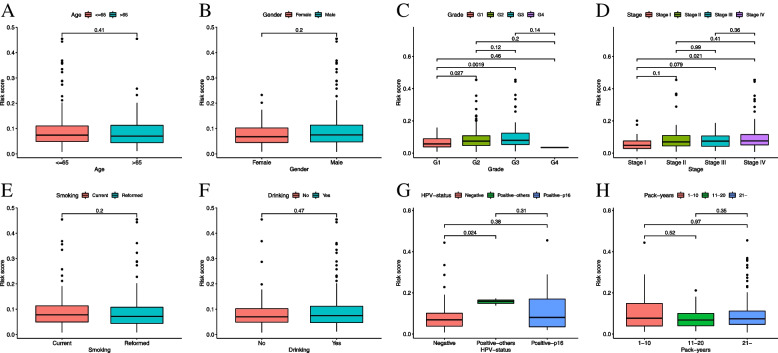
Relationship between prognostic signature expression and clinicopathological factors. Correlation between risk score and **A** age; **B** gender; **C** grade; **D** stage; **E** smoking status; **F** alcohol consumption; **G** HPV status, and **H** smoking package years

We evaluated the expression of the signature between clinical groups. In the smoking population of the HNSCC cohort, high signature expression was observed in patients aged less than or equal to 65 years (*p* = 0.41, Fig. 3A) and males (*p* = 0.20, Fig. 3B). The expression increased significantly with increasing grade (G1-G2: *p* = 0.027; G1-G3: *p* = 0.0019; G2-G3: *p* = 0.12, Fig. 3C) and 7th edition AJCC TNM pathology stage (stage I-stage II: *p* = 0.1; stage I-stage III: *p* = 0.079; stage I-stage IV: *p* = 0.021; stage II-stage III: *p* = 0.99; stage II-stage IV: *p* = 0.41; stage III-stage IV: *p* = 0.36, Fig. 3D), indicating the potential cancer-promoting effects of the signature in HNSCC. Consistent with our results, the expression of nAChRs was high in current smokers as compared to the reformed smokers (*p* = 0.20, Fig. 3E). However, no significant relationship between prognostic signature expression and smoking package years was observed (Fig. 3H). Similar findings were observed between alcohol drinkers and non-drinkers (*p* = 0.47, Fig. 3F). Interestingly, the expression of nAChRs was significantly higher in HPV‐positive smokers with HNSCC, especially with other HPV types, relative to the HPV‐negative smokers (Fig. 3G). We also performed a Kaplan–Meier survival analysis according to stratification by HPV status using the prognostic signature (Supplementary Fig. 1B-C).

### Immune contexts between groups

**Fig. 4:**
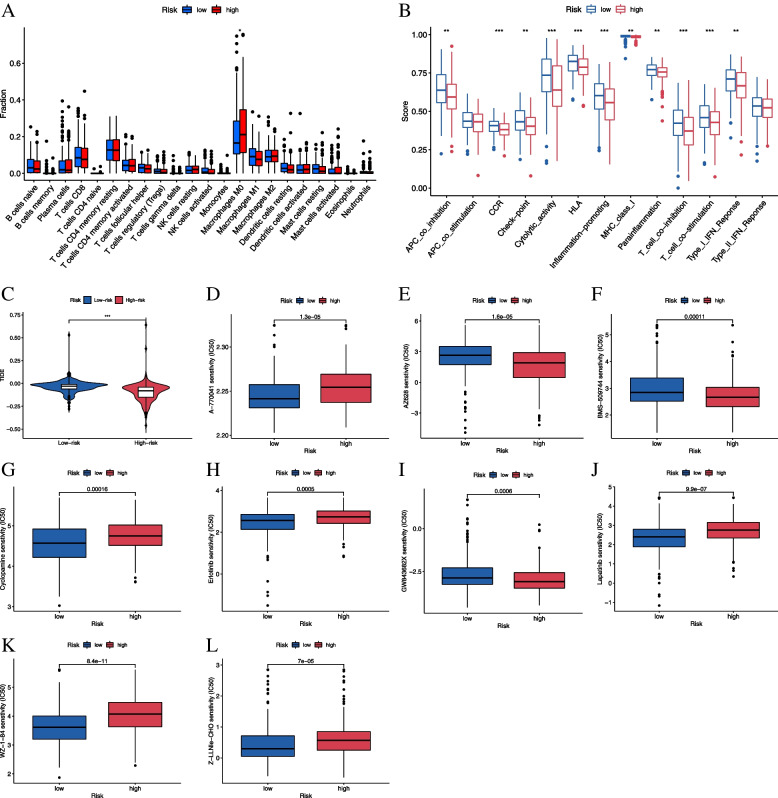
Immune infiltration and drug sensitivity analysis. **A** The proportion of immune cells between low- and high-risk groups based on the CIBERSOR algorithm, wherein the abscissa represents 22 immune cell types while the ordinate represents the proportions of immune cells. *p < 0.05; **B** The immune activities in different groups based on ssGSEA, wherein the abscissa represents 13 immune-related functions while the ordinate represents the ssGSEA scores, **p < 0.01, ***p < 0.001; **C** TIDE scores for different groups; Sensitivity between low- (blue box) and high-risk (red box) groups for **D** A-770041 **E** AZ628; **F** BMS-509744; **G** cyclopamine; **H** erlotinib; **I** GW843682X; **J** lapatinib; **K** WZ-1–84, and **L** Z-LLNIe-CHO

The impact of smoking use on the immune microenvironment is well recognized. As part of our analyses, two algorithms were used to characterize the immune microenvironment between low- and high-risk groups. Through the CIBERSORT algorithm, macrophage M0 was found to be highly enriched in the high-risk group (*p* < 0.05, Fig. 4A). Based on the ssGSEA, the high-risk group was found to show weak immunoreactivity (Fig. 4B), which could account for the poor outcomes of the patients in this group. Based on low TIDE scores, we speculated that the patients in the high-risk group may be more sensitive to immunotherapy (Fig. 4C). For immunotherapy, using the pRRophetic algorithm, nine potential drugs specifically targeting the smoking population in the HNSCC cohort were screened (Fig. 4D-L).

### The nAChR-based prognostic signature is an independent prognostic factor for the smoking population in the HNSCC cohort

**Fig. 5:**
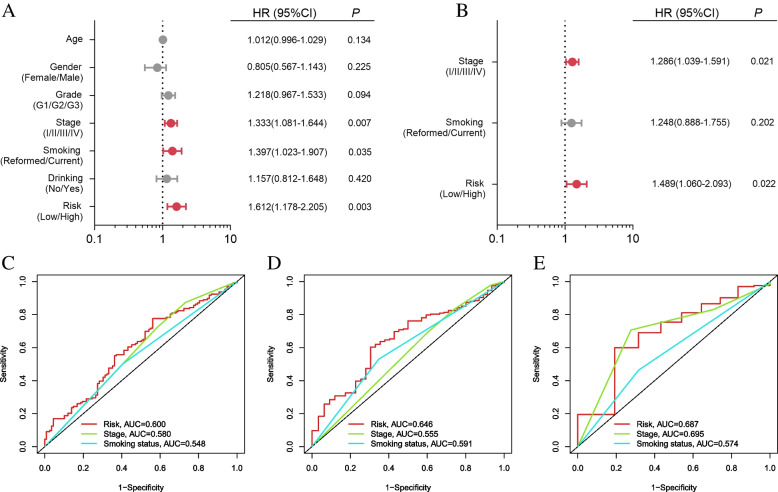
Analysis of independent prognostic factors. **A** Univariate analysis. The red and gray dots represent the significant and insignificant adverse prognostic factors; **B** Multivariate analysis. The red and gray dots represent the significant and insignificant adverse prognostic factors; ROC curve for **C** three-year OS, **D** five-year OS, and **E** ten-year OS

As shown in Fig. 5A, TNM pathology stage, smoking status, and risk score according to the prognostic signature were robustly related to the prognosis of the smoking population in the HNSCC cohort. Further, TNM pathology stage and risk score were independent prognostic factors for OS (Fig. 5B). Time-dependent AUC values of the three prognostic variables, namely TNM stage, smoking status, and risk score, are shown in Fig. 5C-E and risk score showed better sensitivity and specificity for prognostic prediction as compared to TNM pathology stage and smoking status.

### A nomogram based on nAChR expression for prognostic prediction for the smoking population in the HNSCC cohort

**Fig. 6:**
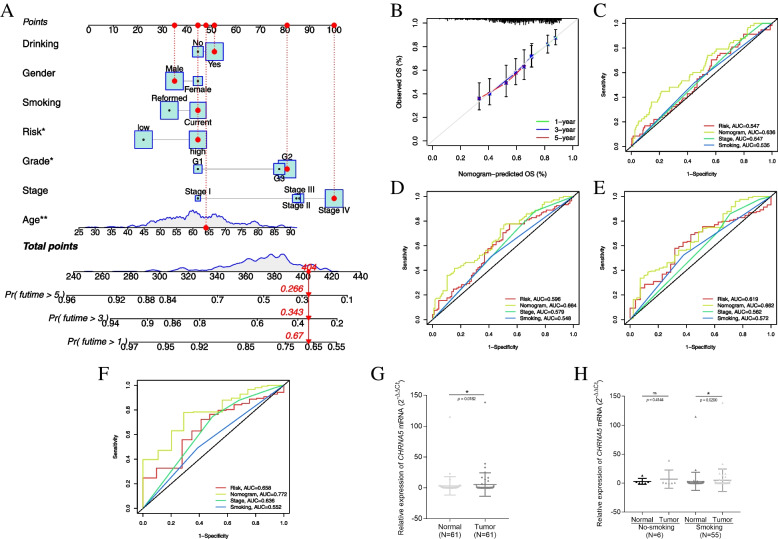
Construction and validation of a nomogram based on the nAChR-based prognostic signature. **A** The nomogram was constructed using clinical variables and nAChR expression. According to the scores for different clinical variables (drinking status, gender, smoking, risk, grade, stage, and age), shown in the upper panel, the survival rate of smoking HNSCC patients was calculated and predicted as shown in the bottom panel; **B** Time-dependent calibration curves for the nomogram. The Grey curve represents the predictive performance of an ideal nomogram while other curves represent the performance of the actual nomogram for one-year OS (green), three-year OS (blue), and five-year OS (red); ROC curves for **C** one-year OS, **D** three-year OS, **E** five-year OS, and **F** seven-year OS; **G** The expression of α5 nAChR in HNSCC samples, *p < 0.05; **H** The expression of α5 nAChR in HNSCC samples with different smoking status, *p < 0.05, ns represents no significance

According to the clinical variables and nAChR expression, we constructed a nomogram for predicting outcomes in the smoking population of the HNSCC cohort in clinical settings (Fig. 6A). For the validation of this nomogram, calibration curves displayed extremely high concordance between the actual observation and the nomogram-based prediction (Fig. 6B), and the time-dependent ROC curves indicated that the nomogram showed higher sensitivity and specificity for predicting one-, three-, five-, and seven-year OS as compared to other prognostic variables (Fig. 6C-F). To demonstrate the additional contribution to predictive accuracy using the nAChR-based signature, we plotted the time-dependent ROC curves without calculating signature expression (Supplementary Fig. 1D-G). For three-, five- and seven-year OS, the AUC values of the prognostic nomogram based on nAChR-associated signature were higher than the null model without calculating the signature expression.

### Validation of nAChR overexpression in the smoking population of the HNSCC cohort

All the above results indicated the critical role of nAChRs in HNSCC patients, especially among smokers. Following qRT-PCR analysis, we selected and tested the mRNA expression of α5 nAChR. Among the 61 paired HNSCC samples, α5 nAChR was highly expressed in HNSCC tissues as compared to the adjacent normal tissues (*p* = 0.0182, Fig. 6G). When stratified according to the smoking status, the same trend was observed in the smoking population (*p* = 0.029) and there was no difference in α5 nAChR expression between tumor and adjacent normal tissues of non-smoking patients (Fig. 6H).

## Discussion

HNSCC is the sixth most common cancer worldwide, and nearly half a million deaths were reported in 2018 due to HNSCC [7]. It is mainly caused by exposure to tobacco through smoking, excessive alcohol consumption, and HPV infection. Despite the rising number of patients with HPV infection, smoking and alcohol abuse remain the most prominent risk factors for HNSCC, especially those originating from the oral cavity and larynx.

The link between smoking and deteriorating health has been confirmed. Annually, millions of premature deaths are caused globally due to smoking [24]. Tobacco comprises several carcinogens that can lead to cancer. Smoking leads to the generation of DNA adducts and mutations in multiple genes [25]. Furthermore, tobacco smoking leads to exposure to carcinogens such as nicotine-derived nitrosamine ketone (NNK) and nicotine, which exert their biological functions by binding to nAChRs [26]. Based on the underlying mechanisms, numerous studies have focused on the role of nAChRs in tumorigenesis and cancer development. In lung cancer, nicotine promotes migration and stemness of non-small cell lung adenocarcinoma (NSCLC) cells via the α7 nAChR/Yap1/E2F1 axis [27]. α5 nAChR contributes to epithelial-mesenchymal transition (EMT) and metastasis of NSCLC cells by mediating the Jab1/Csn5 axis [28]. In head and neck cancer, nicotine causes the phosphorylation of EGFR by regulating α7 nAChR, leading to lymph node metastasis and cetuximab resistance in HNSCC cells [29]. nAChRs may function as oncogenes and mediate oncogenic activities in cancer cells. Consistent with previous studies, nAChRs were highly expressed in head and neck tumors and enriched in neuroactive ligand-receptor interaction, cholinergic synapse, chemical carcinogenesis-receptor activation, and nicotine addiction pathways. The expression of the nAChR-based signature increased significantly with increasing grade and TNM stage, suggesting that it could mediate oncogenic activities in HNSCC.

**Fig. 7:**
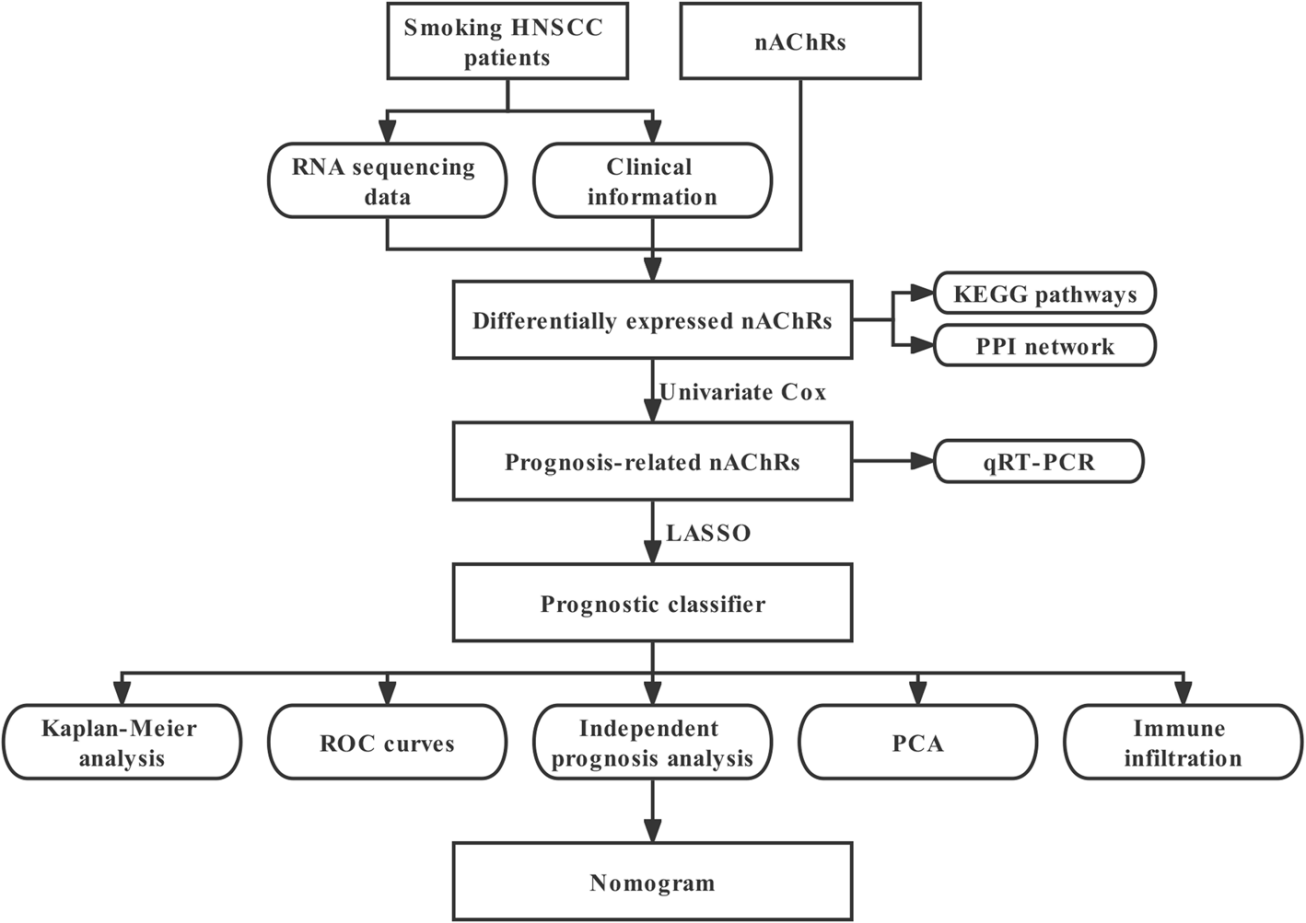
Flowchart of the study

Studies on smoking have mainly focused on lung-related diseases, while related reports on head and neck cancer are scarce. In this present study, for the first time, the expression of nAChR transcripts rather than a single nAChR was used for predicting the outcomes in the smoking population of the HNSCC cohort through bioinformatic analyses and molecular biology techniques. Differential expressed genes may have potential biological function and significance in specific diseases. As is shown in Fig. 7, we firstly identified differentially expressed nAChRs (α5, α6, α9, β2, β3 and β4) via Wilcoxon Rank-Sum Test. Meanwhile, genes associated with prognosis may have the potential to be effective biomarkers for a certain disease. Therefore, we further screened out prognosis-related nAChRs (α2, α5, α9, and β4) via univariate Cox regression analysis. Finally, least absolute shrinkage and selection operator (LASSO) analyses was performed to avoid overfitting in model calculations, α2 nAChR was filtered out and the other three nAChRs (α5, α9, and β4) were included in the prognostic signature which showed a good performance. Although the AUC value of the prognostic signature in the smoking population of the HNSCC cohort was low, the nAChRs-based signature could distinguish the patients into two groups with significant differences in OS and PFS (Fig. 2A-B) and showed an excellent clustering ability (Fig. 2C).

As the most studied nAChR, the cholinergic receptor nicotinic alpha 5 subunit (α5 nAChR), encodes CHRNA5 protein and mediates ligand-gated ion channels when it is connected to the α3 and β4 subunits [30]. Some studies suggest that α5 nAChR promotes cell migration and invasion in lung cancer [28, 31], prostate cancer [32], and breast cancer [33]. Mechanistically, α5 nAChR mediates the Stat3-Jab1/Csn5 and TGF-β1/Smad signaling pathways to promote metastasis and EMT of lung cancer cells [28, 31]. Further, α5 nAChR activates AKT signaling, which in turn promotes metastasis and proliferation of prostate cancer cells [32]. α5 nAChR also promotes metastasis and stemness of hepatocellular carcinoma cells by regulating the Hippo signaling pathway [34]. Cholinergic receptor nicotinic alpha 9 subunit (α9 nAChR) is predominantly expressed in the outer hair cells of the cochlea [35], which plays a critical role in the regulation of the proteins of deafness genes. The expression of α9 nAChR is related to the regulation of pain [36] and polymorphisms in this gene increase the risk of lung cancer [37] and breast cancer [38]. For β4 nAChR, most studies have focused on the CHRNA5/CHRNA3/CHRNB4 cluster, wherein polymorphisms may be associated with an increased risk of death and incidence of cancer among smokers [39–43]. We performed a survival analysis based on the expression of α5, α9, and β4 nAChRs in all HNSCC patients using the Kmplot database. As shown in Supplementary Fig. 2A-C, patients with high α5 nAChR expression showed a significantly poorer OS (HR = 1.54, log-rank *p* = 0.0018); patients with high β4 nAChR expression showed a significantly poorer OS (HR = 1.51, log-rank *p* = 0.0076), and patients with high α9 nAChR expression showed a poorer OS (HR = 1.11, log-rank *p* = 0.44). In addition, methylated sites of α5, α9, and β4 nAChRs in all HNSCC patients were investigated using the MethSurv database as shown in Supplementary Fig. 2D-F.

In this study, α5, α9, and β4 nAChRs were highly expressed in HNSCC tissues and significantly related to poor outcomes in the smoking population of the HNSCC-TCGA cohort. Another cohort comprising 61 paired HNSCC samples showed high levels of α5 nAChR in HNSCC tissues as compared to the adjacent normal tissues, and differential expression was observed in 55 paired samples from patients who were smokers. These findings may shed light on the molecular mechanism underlying nAChRs among smokers with HNSCC.

Accumulating evidence suggests that HPV is critical for HNSCC, especially oropharyngeal cancer [44]. The major understanding of this relationship has come from studies on cervical cancer, a female-specific tumor closely related to HPV. However, different studies have shed different perspectives on this relationship. In this study, we observed that smoking-mediated nAChR expression was different among groups with distinct HPV infection statuses. Patients with other HPV infections showed the highest expression of nAChRs, whereas HPV^+^ p16^+^ HNSCC patients exhibited higher levels of nAChRs relative to HPV^−^ HNSCC patients (Fig. 3G).

Smoking interferes with the immune system and depresses functional immune activity [45]. Moreover, sustained smoking induces autoimmunity to self-antigens, which may cause a range of deleterious effects on smokers or the people around them [45]. High-risk patients with higher nAChR expression showed weaker immunoreactivity than the low-risk patients. These findings may explain the reason for poor prognoses of patients at high risk. We performed immune infiltration analysis based on the expression of α5, α9, and β4 nAChRs in HNSCC patients using the Tumor IMmune Estimation Resource (TIMER) database. As shown in Supplementary Fig. 2G-I, CHRNA9 correlated negatively with B cell (*p* = 1.94e-03) and CD8 + T cell (*p* = 3.27e-06) infiltration. CHRNB4 correlated positively with B cell (*p* = 9.40e-04), CD8 + T cell (*p* = 2.86e-02), CD4 + T cell (*p* = 7.26e-06), macrophage (*p* = 7.96e-04), and dendritic cell (*p* = 1.23e-03) infiltration. In the future, studies should focus on the role of smoking in the immune system of patients with head and neck cancer, which could contribute to the discovery and development of immune checkpoint-related drugs.

Our study, however, has some limitations. For clinical utility and translation, validation of the nAChR-based signature in independent HNSCC cohorts with a smoking population is necessitated. Silencing or overexpression of nAChRs both in vitro and in vivo should be performed to identify hub phenotypic features for head and neck cancer. The potential downstream mechanisms should be elucidated using protein mass spectrometry and/or transcriptome sequencing technologies following the manipulation of levels of nAChR expression.

## Conclusion

In this study, we elucidated the role of nAChRs in HNSCC and constructed a nAChR-based signature that showed a better classification of the smoking population in the HNSCC cohort.

## Supplementary Information

**Additional file 1: Supplementary Figure 1. **Prognosticanalysis of the nAChR-based prognostic signature. (A) Expression profile of β3nAChR; blue and red dots represent normal adjacent and tumor tissues,respectively. **p < 0.01; (B) Kaplan-Meier survivalanalysis of HPV^-^ HNSCC patients using the prognostic signature; (C) Kaplan-Meier survivalanalysis of HPV^+^ HNSCC patients using the prognostic signature; ROCcurves without calculating signature expression for (D) one-year OS, (E) three-year OS, (F) five-year OS, and (G) seven-year OS.

**Additional file 2: Supplementary Figure 2. **Survival analysis, methylated sites and immuneinfiltration analysis of hub nAChRs. Survival analysis based on the expressionof (A) α5, (B)α9, and (C) β4 nAChRs in all HNSCC patients; Methylated sites of(D) α5, (E)α9, and (F) β4 nAChRs in all HNSCC patients; Immune infiltrationanalysis based on the expression of (G) α5, (H)α9, and (I) β4 nAChRs in HNSCCpatients.

**Additional file 3: Supplementary Table 1.** Clinical information of smoking HNSCC cohort.

**Additional file 4: Supplementary Table 2.** Clinical information of adjacent normal samples.

**Additional file 5: Supplementary Table 3.** Clinical information of 61 paired samples diagnosed with HNSCC.

**Additional file 6: Supplementary Table 4.** The sequences of all primers used in this study.

## Data Availability

The datasets analyzed during the current study are publicly available in the TCGA repository (https://portal.gdc.cancer.gov/repository?facetTab=cases; accession date: 12/03/2020; accession number: phs000178).

## Abbreviations

HNSCC: Head and neck squamous cell carcinoma
nAChRs: Nicotinic acetylcholine receptors
TCGA: The Cancer Genome Atlas
LASSO: Least absolute shrinkage and selection operator
KM: Kaplan–Meier
ROC: Receiver operating characteristic
AUC: Area under the curve
PCA: Principal component analysis
HPV: Human papillomavirus
KEGG: Kyoto Encyclopedia of Genes and Genomes
CIBERSORT: Cell type identification by estimating relative subsets of RNA transcripts
ssGSEA: Single-sample Gene Set Enrichment Analysis
TIDE: Tumor Immune Dysfunction and Exclusion
GDSC: Drug Sensitivity in Cancer
qRT-PCR: Quantitative reverse transcriptase PCR
OS: Overall survival
PFS: Progression free survival
TNM: Tumor Node Metastasis
DNA: Deoxyribonucleic acid
NNK: Nicotine-derived nitrosamine ketone
NSCLC: Non-small cell lung adenocarcinoma
Yap1: Yes1 associated transcriptional regulator
E2F1: E2F transcription factor 1
EMT: Epithelial-mesenchymal transition
COPS5: COP9 signalosome subunit 5
EGFR: Epidermal growth factor receptor
CHRNA5: Cholinergic receptor nicotinic alpha 5 subunit
CHRNA3: Cholinergic receptor nicotinic alpha 3 subunit
CHRNB4: Cholinergic receptor nicotinic beta 4 subunit

## References

[1] Johnson DE, Burtness B, Leemans CR, Lui VWY, Bauman JE, Grandis JR (2020). Head and neck squamous cell carcinoma. Nat Rev Dis Primers.

[2] Stein AP, Saha S, Kraninger JL, Swick AD, Yu M, Lambert PF, Kimple RJ (2015). Prevalence of human papillomavirus in oropharyngeal cancer: a systematic review. Cancer J.

[3] Hashibe M, Brennan P, Chuang S-C, Boccia S, Castellsague X, Chen C, Curado MP, Dal Maso L, Daudt AW, Fabianova E (2009). Interaction between tobacco and alcohol use and the risk of head and neck cancer: pooled analysis in the International Head and Neck Cancer Epidemiology Consortium. Cancer Epidemiol Biomarkers Prev.

[4] Chang ET, Liu Z, Hildesheim A, Liu Q, Cai Y, Zhang Z, Chen G, Xie S-H, Cao S-M, Shao J-Y (2017). Active and passive smoking and risk of nasopharyngeal carcinoma: a population-based case-control study in Southern China. Am J Epidemiol.

[5] Whiteman DC, Wilson LF (2016). The fractions of cancer attributable to modifiable factors: a global review. Cancer Epidemiol.

[6] Kaidar-Person O, Gil Z, Billan S (2018). Precision medicine in head and neck cancer. Drug Resist Updat.

[7] Ferlay J, Colombet M, Soerjomataram I, Mathers C, Parkin DM, Piñeros M, Znaor A, Bray F (2019). Estimating the global cancer incidence and mortality in 2018: GLOBOCAN sources and methods. Int J Cancer.

[8] Zoli M, Pucci S, Vilella A, Gotti C (2018). Neuronal and extraneuronal nicotinic acetylcholine receptors. Curr Neuropharmacol.

[9] Russo P, Cardinale A, Margaritora S, Cesario A (2012). Nicotinic receptor and tobacco-related cancer. Life Sci.

[10] Millar NS, Gotti C (2009). Diversity of vertebrate nicotinic acetylcholine receptors. Neuropharmacology.

[11] Liu W, Li MD (2018). Insights into nicotinic receptor signaling in nicotine addiction: implications for prevention and treatment. Curr Neuropharmacol.

[12] Blum A, Wang P, Zenklusen JC (2018). SnapShot: TCGA-analyzed tumors. Cell.

[13] von Mering C, Huynen M, Jaeggi D, Schmidt S, Bork P, Snel B (2003). STRING: a database of predicted functional associations between proteins. Nucleic Acids Res.

[14] Kanehisa M, Furumichi M, Sato Y, Ishiguro-Watanabe M, Tanabe M (2021). KEGG: integrating viruses and cellular organisms. Nucleic Acids Res.

[15] Zhou L, Yu Y, Wen R, Zheng K, Jiang S, Zhu X, Sui J, Gong H, Lou Z, Hao L (2022). Development and validation of an 8-gene signature to improve survival prediction of colorectal cancer. Front Oncol.

[16] Kim Y, Kang JW, Kang J, Kwon EJ, Ha M, Kim YK, Lee H, Rhee J-K, Kim YH (2021). Novel deep learning-based survival prediction for oral cancer by analyzing tumor-infiltrating lymphocyte profiles through CIBERSORT. Oncoimmunology.

[17] Xu Q, Chen S, Hu Y, Huang W (2021). Landscape of immune microenvironment under immune cell infiltration pattern in breast cancer. Front Immunol.

[18] Yang W, Soares J, Greninger P, Edelman EJ, Lightfoot H, Forbes S, Bindal N, Beare D, Smith JA, Thompson IR (2013). Genomics of Drug Sensitivity in Cancer (GDSC): a resource for therapeutic biomarker discovery in cancer cells. Nucleic Acids Res.

[19] Iasonos A, Schrag D, Raj GV, Panageas KS (2008). How to build and interpret a nomogram for cancer prognosis. J Clin Oncol.

[20] Huang Q, Shen Y-J, Hsueh C-Y, Guo Y, Zhang Y-F, Li J-Y, Zhou L (2021). miR-17-5p drives G2/M-phase accumulation by directly targeting CCNG2 and is related to recurrence of head and neck squamous cell carcinoma. BMC Cancer.

[21] Lánczky A, Győrffy B (2021). Web-based survival analysis tool tailored for medical research (KMplot): development and implementation. J Med Internet Res.

[22] Modhukur V, Iljasenko T, Metsalu T, Lokk K, Laisk-Podar T, Vilo J (2018). MethSurv: a web tool to perform multivariable survival analysis using DNA methylation data. Epigenomics.

[23] Li T, Fu J, Zeng Z, Cohen D, Li J, Chen Q, Li B, Liu XS (2020). TIMER2.0 for analysis of tumor-infiltrating immune cells. Nucleic Acids Res.

[24] Kalkhoran S, Glantz SA (2016). E-cigarettes and smoking cessation in real-world and clinical settings: a systematic review and meta-analysis. Lancet Respir Med.

[25] Hecht SS, Hatsukami DK (2022). Smokeless tobacco and cigarette smoking: chemical mechanisms and cancer prevention. Nat Rev Cancer.

[26] Picciotto MR, Kenny PJ (2021). Mechanisms of nicotine addiction. Cold Spring Harb Perspect Med.

[27] Schaal CM, Bora-Singhal N, Kumar DM, Chellappan SP (2018). Regulation of Sox2 and stemness by nicotine and electronic-cigarettes in non-small cell lung cancer. Mol Cancer.

[28] Chen X, Jia Y, Zhang Y, Zhou D, Sun H, Ma X (2020). α5-nAChR contributes to epithelial-mesenchymal transition and metastasis by regulating Jab1/Csn5 signalling in lung cancer. J Cell Mol Med.

[29] Shimizu R, Ibaragi S, Eguchi T, Kuwajima D, Kodama S, Nishioka T, Okui T, Obata K, Takabatake K, Kawai H (2019). Nicotine promotes lymph node metastasis and cetuximab resistance in head and neck squamous cell carcinoma. Int J Oncol.

[30] Hurst R, Rollema H, Bertrand D (2013). Nicotinic acetylcholine receptors: from basic science to therapeutics. Pharmacol Ther.

[31] Zhang Q, Jia Y, Pan P, Zhang X, Jia Y, Zhu P, Chen X, Jiao Y, Kang G, Zhang L, et al. α5-nAChR associated with Ly6E modulates cell migration via TGF-β1/Smad signaling in non-small cell lung cancer. Carcinogenesis. 2022:bgac003. 10.1093/carcin/bgac003 10.1093/carcin/bgac003 34994389

[32] Qi J-C, Xue W-Y, Zhang Y-P, Qu C-B, Lu B-S, Yin Y-W, Liu K-L, Wang D-B, Li W, Zhao Z-M (2020). Cholinergic α5 nicotinic receptor is involved in the proliferation and invasion of human prostate cancer cells. Oncol Rep.

[33] Shehwana H, Keskus AG, Ozdemir SE, Acikgöz AA, Biyik-Sit R, Cagnan I, Gunes D, Jahja E, Cingir-Koker S, Olmezer G (2021). CHRNA5 belongs to the secondary estrogen signaling network exhibiting prognostic significance in breast cancer. Cell Oncol (Dordr).

[34] Fu Y, Ci H, Du W, Dong Q, Jia H (2022). CHRNA5 Contributes to Hepatocellular Carcinoma Progression by Regulating YAP Activity. Pharmaceutics.

[35] Elgoyhen AB, Johnson DS, Boulter J, Vetter DE, Heinemann S (1994). Alpha 9: an acetylcholine receptor with novel pharmacological properties expressed in rat cochlear hair cells. Cell.

[36] Hone AJ, Servent D, McIntosh JM (2018). α9-containing nicotinic acetylcholine receptors and the modulation of pain. Br J Pharmacol.

[37] Wang Y, Zhang Y, Gu C, Bao W, Bao Y (2014). Neuronal acetylcholine receptor subunit alpha-9 (CHRNA9) polymorphisms are associated with NSCLC risk in a Chinese population. Med Oncol.

[38] Hsieh Y-C, Lee C-H, Tu S-H, Wu C-H, Hung C-S, Hsieh M-C, Chuang C-W, Ho Y-S, Chiou H-Y (2014). CHRNA9 polymorphisms and smoking exposure synergize to increase the risk of breast cancer in Taiwan. Carcinogenesis.

[39] Zhao Z, Peng F, Zhou Y, Hu G, He H, He F, Zou W, Zhao Z, Li B, Ran P (2015). Exon sequencing identifies a novel CHRNA3-CHRNA5-CHRNB4 variant that increases the risk for chronic obstructive pulmonary disease. Respirology.

[40] Lee S-H, Ahn W-Y, Seweryn M, Sadee W (2018). Combined genetic influence of the nicotinic receptor gene cluster CHRNA5/A3/B4 on nicotine dependence. BMC Genomics.

[41] Barrie ES, Hartmann K, Lee S-H, Frater JT, Seweryn M, Wang D, Sadee W (2017). The CHRNA5/CHRNA3/CHRNB4 nicotinic receptor regulome: genomic architecture, regulatory variants, and clinical associations. Hum Mutat.

[42] Wen L, Jiang K, Yuan W, Cui W, Li MD (2016). Contribution of Variants in CHRNA5/A3/B4 gene cluster on chromosome 15 to tobacco smoking: from genetic association to mechanism. Mol Neurobiol.

[43] Halldén S, Sjögren M, Hedblad B, Engström G, Hamrefors V, Manjer J, Melander O (2016). Gene variance in the nicotinic receptor cluster (CHRNA5-CHRNA3-CHRNB4) predicts death from cardiopulmonary disease and cancer in smokers. J Intern Med.

[44] Leemans CR, Snijders PJF, Brakenhoff RH (2018). The molecular landscape of head and neck cancer. Nat Rev Cancer.

[45] Lee J, Taneja V, Vassallo R (2012). Cigarette smoking and inflammation: cellular and molecular mechanisms. J Dent Res.

