# Supplementary material for: Smoking-mediated nicotinic acetylcholine receptors (nAChRs) for predicting outcomes for head and neck squamous cell carcinomas
Source: BMC Cancer. 2022 Oct 25;22:1093. doi: 10.1186/s12885-022-10161-x (PMC9594873; doi:10.1186/s12885-022-10161-x)
Supplement: Supplementary file 1 — Additional file 1: Supplementary Figure 1. Prognosticanalysis of the nAChR-based prognostic signature. (A) Expression profile of β3nAChR; blue and red dots represent normal adjacent and tumor tissues,respectively. **p < 0.01; (B) Kaplan-Meier survivalanalysis of HPV- HNSCC patients using the prognostic signature; (C) Kaplan-Meier survivalanalysis of HPV+ HNSCC patients using the prognostic signature; ROCcurves without calculating signature expression for (D) one-year OS, (E) three-year OS, (F) five-year OS, and (G) seven-year OS. [file 12885_2022_10161_MOESM1_ESM.docx]

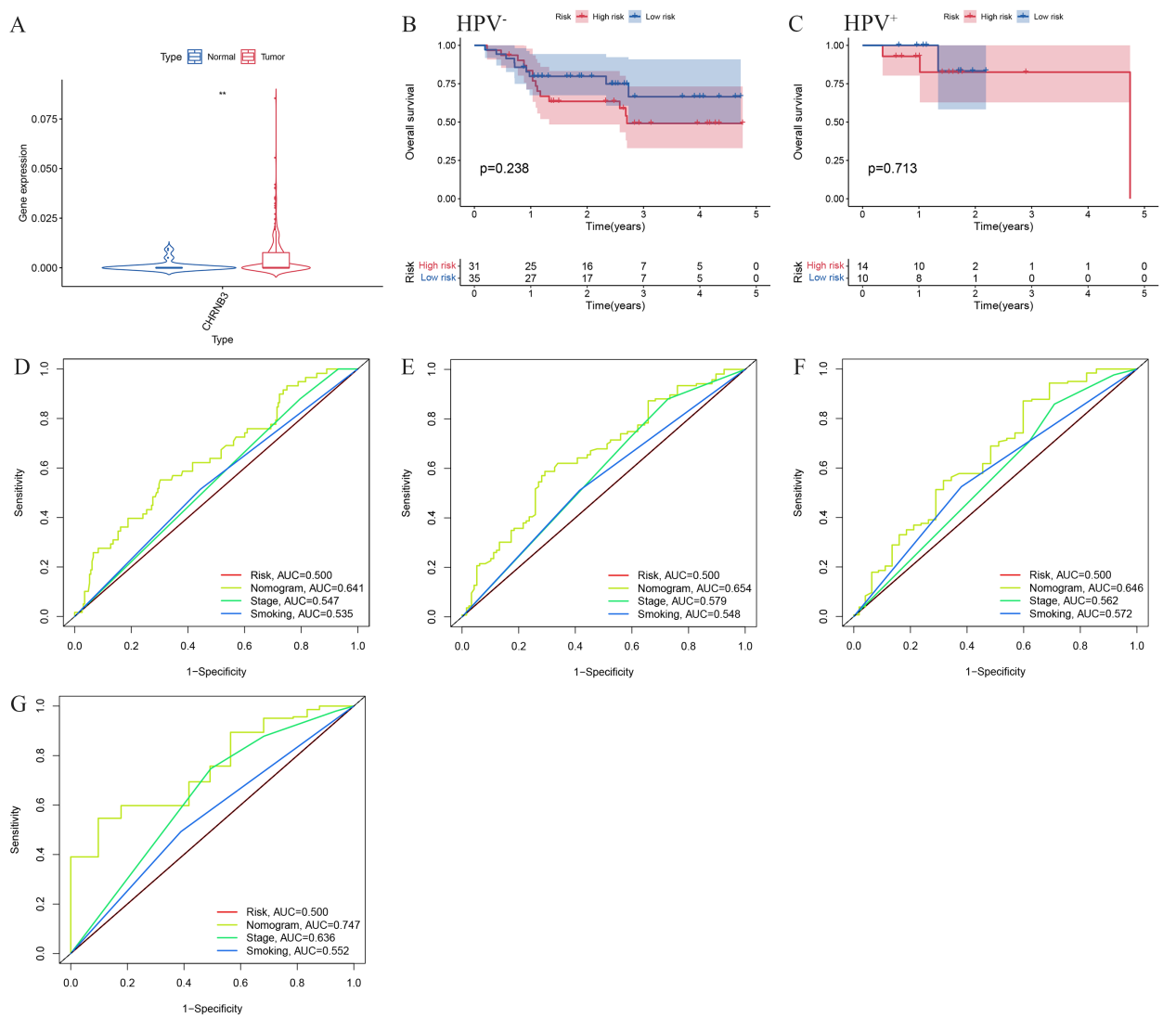


**Supplementary Figure 1:** Prognostic analysis of the nAChR-based prognostic signature. (A) Expression profile of β3 nAChR; blue and red dots represent normal adjacent and tumor tissues, respectively. ***p* < 0.01; (B) Kaplan-Meier survival analysis of HPV^-^ HNSCC patients using the prognostic signature; (C) Kaplan-Meier survival analysis of HPV^+^ HNSCC patients using the prognostic signature; ROC curves without calculating signature expression for (D) one-year OS, (E) three-year OS, (F) five-year OS, and (G) seven-year OS.
