# Supplementary material for: Smoking-mediated nicotinic acetylcholine receptors (nAChRs) for predicting outcomes for head and neck squamous cell carcinomas
Source: BMC Cancer. 2022 Oct 25;22:1093. doi: 10.1186/s12885-022-10161-x (PMC9594873; doi:10.1186/s12885-022-10161-x)
Supplement: Supplementary file 2 — Additional file 2: Supplementary Figure 2. Survival analysis, methylated sites and immuneinfiltration analysis of hub nAChRs. Survival analysis based on the expressionof (A) α5, (B)α9, and (C) β4 nAChRs in all HNSCC patients; Methylated sites of(D) α5, (E)α9, and (F) β4 nAChRs in all HNSCC patients; Immune infiltrationanalysis based on the expression of (G) α5, (H)α9, and (I) β4 nAChRs in HNSCCpatients. [file 12885_2022_10161_MOESM2_ESM.docx]

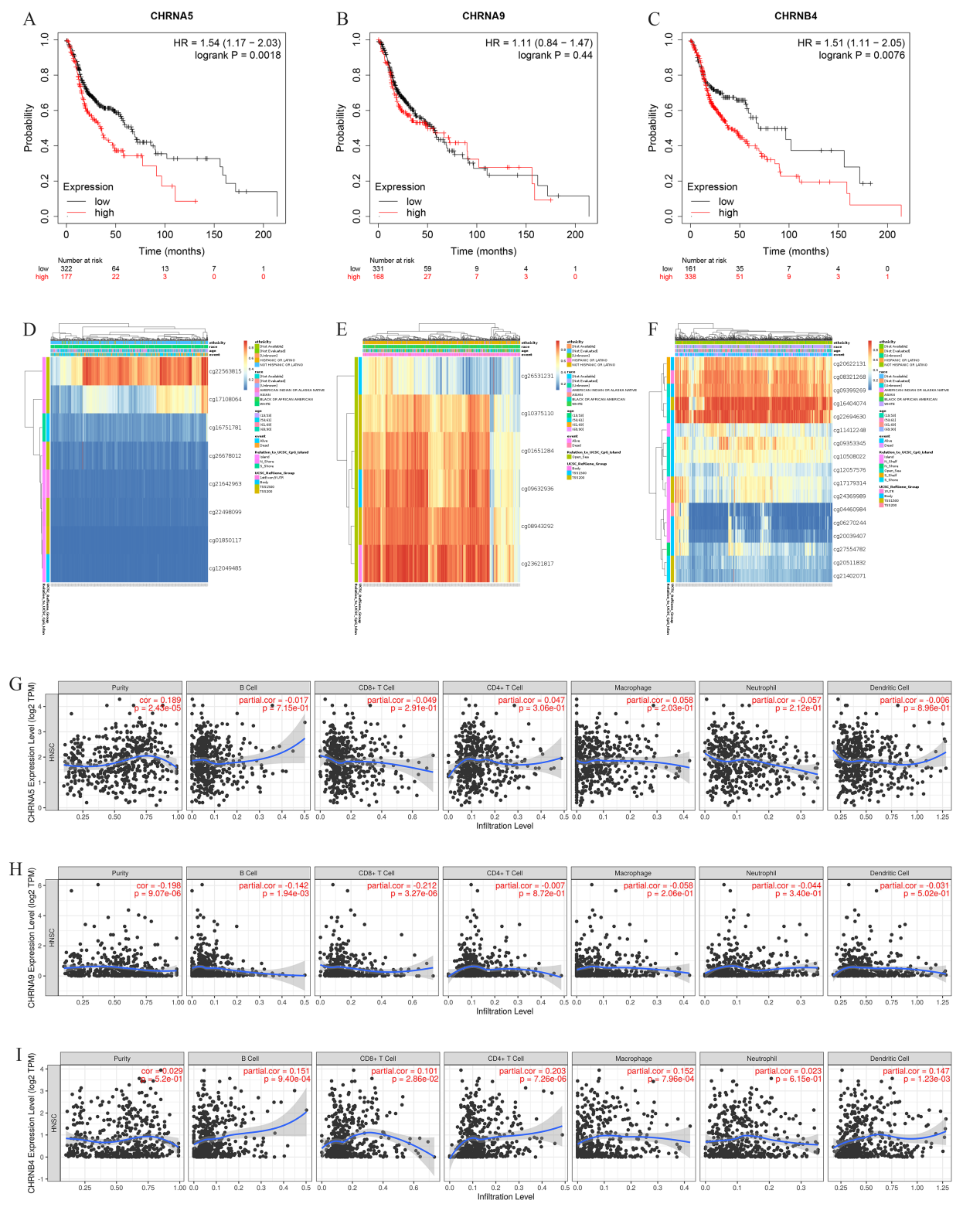


**Supplementary Figure 2:** Survival analysis, methylated sites and immune infiltration analysis of hub nAChRs. Survival analysis based on the expression of (A) α5, (B)α9, and (C) β4 nAChRs in all HNSCC patients; Methylated sites of (D) α5, (E)α9, and (F) β4 nAChRs in all HNSCC patients; Immune infiltration analysis based on the expression of (G) α5, (H)α9, and (I) β4 nAChRs in HNSCC patients.
